# Draft Genome Sequences of Nine “*Candidatus* Nanosynbacter sp. HMT-352” Strains Cultured from the Human Oral Cavity

**DOI:** 10.1128/mra.00403-22

**Published:** 2022-07-27

**Authors:** Jett Liu, Daniel R. Utter, Jie Nie, Kristopher A. Kerns, Eleanor I. Lamont, Erik L. Hendrickson, Xiaoyan Wang, Jeffrey S. McLean, Xuesong He, Batbileg Bor

**Affiliations:** a The Forsyth Institute, Cambridge, Massachusetts, USA; b Department of Organismic and Evolutionary Biology, Harvard University, Cambridge, Massachusetts, USA; c Department of Cariology and Endodontology, Peking University School of Stomatology, Beijing, China; d Department of Periodontics, University of Washington, Seattle, Washington, USA; e Department of Oral Medicine, Infection, and Immunity, Harvard School of Dental Medicine, Boston, Massachusetts, USA; Indiana University, Bloomington

## Abstract

Here, we report draft genome sequences for nine strains of “*Candidatus* Nanosynbacter sp. HMT-352.” These strains and their sequences were used to interrogate strain-level variations in host range, gene content, and growth dynamics among the phylum “*Candidatus* Saccharibacteria.”

## ANNOUNCEMENT

The nine “*Candidatus* Nanosynbacter sp. HMT-352” (hereafter, HMT-352) strains reported here (Table 1) were recently isolated from the human oral cavity (1) and are the first members of the phylum “*Candidatus* Saccharibacteria,” a major lineage of the Candidate Phyla Radiation (CPR) (2), to be characterized at the strain level.

**TABLE 1:** Summary information for the nine “*Ca*. Nanosynbacter sp. HMT-352” strains cultured from the human oral cavity

Strain name	BioSample accession no.	SRA accession no. for:		Total length (bp)	No. of contigs	*N*_50_ (bp)	GC content (%)	Completion (%)	Redundancy (%)	No. of genes	Assembly coverage (×) for:	
		Monoculture	Coculture								Monoculture	Coculture
TM7-001	SAMN23492223	SRR18278454	SRR18278446	771,807	1	771,807	43.25	83.10	2.82	803	3,276	432
TM7-008	SAMN23492221	SRR18278459	SRR18278451	725,580	2	548,424	43.17	84.51	0.00	747	3,063	2,387
TM7-053	SAMN23492224	SRR18278461	SRR18278463	755,984	2	546,117	43.18	84.51	4.23	775	1,403	1,097
TM7-057	SAMN23492218	SRR18278457	SRR18278449	758,480	6	490,402	42.99	84.51	2.82	804	728	1,156
TM7-072	SAMN23492220	SRR18278453	SRR18278445	733,210	1	733,210	43.31	84.51	1.41	742	2,911	1,235
TM7-075	SAMN23492222	SRR18278460	SRR18278462	730,938	2	448,955	43.15	84.51	4.23	755	3,484	1,767
TM7-076	SAMN23492217	SRR18278456	SRR18278448	756,098	4	170,979	43.19	83.10	0.00	791	3,519	1,213
TM7-087	SAMN23492219	SRR18278458	SRR18278450	741,912	3	536,841	43.42	84.51	0.00	771	3,259	2,736
TM7-037	SAMN23492216	SRR18278452	SRR18278444	718,283	1	718,283	43.26	83.10	2.82	735	651	390

These nine HMT-352 strains were isolated from human saliva using a previously described “baiting” method (1, 3). Briefly, saliva samples were centrifuged, filtered through a 0.45-μM filter, and cocultured in brain heart infusion medium (catalog number 237500; BD, NJ, USA) with potential basibionts (bacterial hosts). The cocultures were incubated at 37°C and passaged at a dilution of 1:10 every 2 days into fresh medium. A previously described modified MasterPure DNA isolation kit (catalog number MGP04100; Epicentre, WI, USA) protocol (4) was used to isolate genomic DNA (gDNA) from both filter-isolated HMT-352 cells and the HMT-352-basibiont cocultures. Briefly, bacterial cultures were mixed with glass beads (catalog number G8772; Sigma, St. Louis, MO) and disrupted using a bead-beating homogenizer. gDNA isolation was then performed according to the manufacturer’s protocol. The gDNA was randomly fragmented by sonication and then end-polished, A-tailed, and ligated with full-length Illumina adapters. The library constructs were purified using the AMPure XP system (Beckman Coulter, IN, USA) and checked for size distribution using a 2100 Bioanalyzer (Agilent Technologies, CA, USA). The libraries were then sequenced on an Illumina NovaSeq instrument (paired-end [PE] 150-bp reads).

Default parameters were used for the computational analyses except where otherwise noted. The reads were quality controlled using iu-filter-quality-minoche from illumina-utils v2.12 (5). For each new HMT-352 strain, genomes were assembled using a previously described Anvi’o v7.1 workflow (1, 6) that employed both the isolate and coculture genomic libraries. Briefly, for each strain, libraries from the isolated HMT-352 were individually assembled using metaSPAdes v3.15.3 (7) and binned using MaxBin2 v2.2.4-1 (8). The bins were then manually refined and reassembled using both the isolate and coculture libraries. Genes were annotated using the NCBI Prokaryotic Genome Annotation Pipeline (PGAP) v5.3 (9). All genomes were less than 5% redundant, between 83% and 85% complete, and contained between 732 and 801 genes (Table 1).

**FIG 1:**
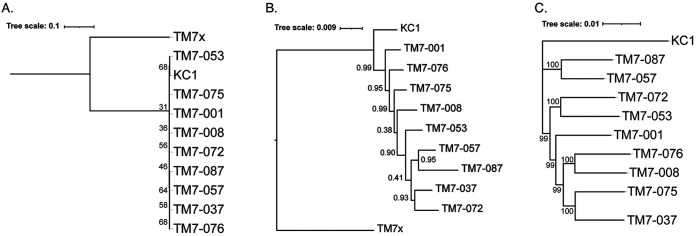
16S rRNA, select marker gene, and single-copy core gene trees of the reported HMT-352 strains. The previously reported HMT-352 strain KC1 (15) is additionally included. The node labels represent bootstrap support. (A) Maximum-likelihood tree based on full-length 16S rRNA sequences constructed using IQ-TREE v2.1.4-beta with ultrafast bootstrap (-bb 1500) (16). *Nanosynbacter lyticus* strain TM7x (17) is included as an outgroup. (B) Phylogenomic tree constructed using FastTree 2 v2.1.11-1 (18) with 60 concatenated core protein amino acid sequences and TM7x (HMT-952) included as an outgroup. (C) Maximum-likelihood tree inferred using 523 concatenated single-copy core gene amino acid sequences found in all strains constructed using IQ-TREE with ultrafast bootstrap (-bb 1500).

The HMT-352 strains were compared to representative *Candidatus* Saccharibacteria from eHOMD v15.22 (10) using full-length 16S rRNA sequences aligned with MAFFT v7.490 (11). All strains had more than 98% homology to the closest eHOMD species. The average nucleotide identity (ANI) values over all alignable genome fractions, however, ranged between 93% and 95%, at or below the extreme end of the accepted range for intraspecies variation (12–14). Such substantial intraspecies genetic diversity is additionally apparent in Fig. 1, which provides a comparison of the phylogenetic differences between the 16S rRNA, select marker gene, and single-copy core gene trees. The unexpectedly high nucleotide diversity among these strains warrants further investigation and accentuates that broad phylogenetic characterization of the CPR is the next step in understanding these bacteria.

### Data availability.

Cultures of these strains are available upon request. The sequence data have been deposited at NCBI under the BioProject accession number PRJNA784561. The BioSample and SRA accession numbers are listed in Table 1. All code used to assemble and analyze the genomes is available at https://www.borlab.org/resources.
